# Regulation of electrolyte transport with IL-1β in rabbit distal colon

**DOI:** 10.1155/S0962935195000111

**Published:** 1995-01

**Authors:** F. R. Homaidan, H. Desai, L. Zhao, G. Broutman, R. Burakoff

**Affiliations:** 1 Division of Gastroenterology and Nutrition Winthrop-University Hospital Mineola NY 11501 USA; 2 Department of Medicine SUNY at Stony Brook Mineola NY 11501 USA

## Abstract

Interletrkin-1β levels are elevated in inflammatory bowel disease. In this study the mechanism by which interleukin-1β affects electrolyte transport in the rabbit distal colon, was investigated. Interleukin-1β caused a delayed increase in short-circuit current (I_sc_) which was attributed to protein synthesis since the effect was inhibited by cycloheximide. The interleukin-1β induced increase in I_sc_ was not affected by amiloride treatment but was completely inhibited by bumetanide or in chloride-free buffer and by indomethacin. Prostaglandin E_2_ levels increased in tissue treated with interleukin-1β, but this increase was reversed by cycloheximide. These data suggest that interleukin-1β causes its effect via a yet to be identified second messenger, by increasing chloride secretion through a prostaglandin E_2_ mediated mechanism.


Research Paper

Mediators of Inflammation 4, 61–66 (1995)

### Regulation of electrolyte transport with IL-1β in rabbit distal colon

F. R. Homaidan,[1,2,CA] H. Desai,[1] L. Zhao,[1] G. Broutman[1] and R. Burakoff[1,2]

[1]Division of Gastroenterology and Nutrition, Winthrop-University Hospital and [2]Department of Medicine, SUNY at Stony Brook, Mineola, NY 11501, USA

[CA] Corresponding Author

**INTERLEUKIN-1β levels are elevated in inflammatory bowel disease. In this study the mechanism by which interleukin-1β affects electrolyte transport in the rabbit distal colon, was investigated. Interleukin-1β caused a delayed increase in short-circuit current ($I_{sc}$) which was attributed to protein synthesis since the effect was inhibited by cycloheximide. The interleukin-1β induced increase in $I_{sc}$ was not affected by amiloride treatment but was completely inhibited by bumetanide or in chloride-free buffer and by indomethacin. Prostaglandin $E_2$ levels increased in tissue treated with interleukin-1β, but this increase was reversed by cycloheximide. These data suggest that interleukin-1β causes its effect via a yet to be identified second messenger, by increasing chloride secretion through a prostaglandin $E_2$ mediated mechanism.**

**Key words**: Chloride secretion, Cytokines, Prostaglandin $E_2$

### Introduction

IL-1 is a cytokine of around 17 kDa which is responsible for mediating a variety of processes in host defence, inflammation, and response to injury. It is produced by diverse cell types following infection or injury.[1] On the basis of several overlapping activities and similar patterns of production, IL-1 may be grouped with tumour necrosis factor-α (TNFα, and IL-6 as factors mediating common effects.[2,3] It is produced by a variety of cells such as fibroblasts and T and B lymphocytes and consists of two distinct but related molecules, IL-1α and IL-1β[4] which are encoded by separate genes; in most human tissues IL-1β mRNA predominates over IL-1α mRNA. Both forms appear to function by binding to the same membrane-associated receptor. IL-1 appears to be a primary molecule in inflammatory reactions through its induction of other inflammatory metabolites, e.g. it induces production of prostaglandin $E_2$ ($PGE_2$) and phospholipase $A_2$ ($PLA_2$).[5]

In the intestine, increased IL-1 production has been reported in the mucosa of patients with inflammatory bowel disease[6–8] and in intestinal tissue of animal models of colitis[9–11] suggesting that IL-1 may be involved in the pathophysiology of inflammatory bowel disease. IL-1 has been shown to stimulate Cl: and $HCO_3^-$ secretion in the chicken intestine[12] and inhibit $Na^+$ and $Cl^-$ absorption in rabbit ileum,[13] however, the role and mechanism of action of IL-1 in affecting secretory function in the normal colon has not been fully elucidated.

More than one second messenger seems to be involved in mediating the effects of IL-1. Some of these mediators include nitric oxide (NO), cAMP and phospholipases C and $A_2$.[14,15] In this study, the actions of IL-1β on electrolyte transport in normal rabbit distal colon and the second messenger(s) that might be involved in its mechanism of action are characterized. It was also determined whether TNFα has similar effects to IL-1 on ion transport in the rabbit colon since, although TNFα and IL-1 are biochemically distinct cytokines, they have many related and overlapping biological functions, and they play important and similar roles in many immune responses.[2,3]

### Materials and Methods

Human recombinant IL-1β, atropine, tetrodotoxin, indomethacin, amiloride, NG-1-nitro-L-arginine methyl ester (NAME), bumetanide, cycloheximide, actinomycin D and human recombinant TNFα were obtained from Sigma (St Louis, MO). For some experiments IL-1β was obtained as a gift from Drs P. Smith and J. Lee at Smith Kline and Beecham (Philadelphia, PA).

*Transport studies:* Male New Zealand albino rabbits weighing 2–3 kg were maintained on a standard rabbit chow diet with free access to water. The animals were killed by an overdose of pentobarbital sodium (i.v.). The distal colon was removed and epithelial sheets devoid of serosa and muscularis propria were prepared for transport studies. Colonic mucosa was mounted as a flat sheet between two Lucite modified Ussing chambers having an aperture of 1.13 cm², and oxygenated and maintained at 37°C. Short-circuit current ($I_{sc}$) which is equivalent to the electrical sum of all ion transport processes occurring simultaneously was determined. An automatic voltage clamp (W.P.I., Sarasota, FL), corrected for fluid

© 1995 Rapid Communications of Oxford Ltd resistance between the potential difference sensing bridges, provided continuous short-circuiting of the tissue.

Unless specified, the bathing solution consisted of Krebs–$HCO_3$ (KBS) composed of (in mM): KCl 4.8, $CaCl_2$ 2.5, NaCl 118.1, $NaH_2PO_4$ 1.2, $MgSO_4$ 1.2, $NaHCO_3$ 25, glucose 11, pH 7.4 after gassing with 95% $O_2$/5% $CO_2$. Human recombinant IL-1β was used in this study. It has previously been shown that rabbit and human IL-1 cross-react with an antibody to human IL-1[16] and that rabbit and human IL-1 have approximately 65% homology.[17] Amiloride and bumetanide were dissolved in 100% ethanol and indomethacin dissolved in 10 mM sodium carbonate. All reagents were added to the serosal bathing solution. Ion substitution studies were performed by either replacing $Cl^-$ ions by gluconate ions or replacing $HCO_3$= ions by HEPES solution.

*Measurement of $PGE_2$ levels in tissue treated with IL-1β:* Stripped distal colon mucosal sheets were washed with KBS, placed on ice and cut into small but equal pieces. For all assays, pieces of tissue were transferred to polypropylene vials containing 5 ml of oxygenated KBS at 37°C in a slow shaking water bath. The tissue was continuously oxygenated during the experiment. Aliquots were transferred from the supernatant to Eppendorf tubes at specific times and centrifuged at 4°C, for 2 min at 7 000 *g* in the Eppendorf centrifuge. Samples were kept at –70°C for later determination of $PGE_2$ levels using radioimmunoassay (RIA). Commercially available RIA kits for measurement of $PGE_2$ were used (Advanced Magnetics Inc., MA). The sample was precipitated with cold acetone, centrifuged and the precipitate discarded. Petroleum ether was then added to the supernatant and the aqueous phase separated and acidified to pH 3–4 and then extracted further with ethyl acetate. The sample was lyophilized and reconstituted in a known amount of assay buffer. In brief, the assay involved incubating the sample with the corresponding antiserum overnight at 4°C and then centrifuging for 15 min at 4°C at 1 000 × **g**. The supernatant was then transferred to scintillation vials, scintillant was added and the vial counted in a liquid scintillation counter.

*Statistical analyses:* Statistical analyses were performed with Student's *t*-test for paired and unpaired data; half-maximal and maximal effects of IL-1β on transport were determined by the method of Woolf–Hanes[18] by plotting the concentration/change in $I_{sc}$ *vs.* concentration. In these calculations, the half-maximal concentration and the maximal effect for each experiment were determined, and the results are presented as the means of these ± S.E. Unless specified all results are reported as mean ± S.E.

### Results

*Effects of IL-1β on $I_{sc}$ in rabbit distal colon:* IL-1 (0.1 to 10 ng/ml) was added to the serosal bathing solution and $I_{sc}$ monitored for 60 min. IL-1β caused a significant, concentration dependent increase in $I_{sc}$ (Fig. 1A) with an $EC_{50}$ of 0.2 ng/ml. The peak increase in $I_{sc}$ was 24.1 ± 2.5 μA/cm² (basal $I_{sc}$ was 79.0 ± 13.5 μA/cm²) and was reached 40–50 min after the addition of IL-1β. This increase however was delayed and only observed 10–20 min after the addition of IL-1β (Fig. 1B).

*Effects if IL-1β on $I_{sc}$ in the presence of indomethacin, atropine and TTX:* To determine the mechanism by which IL-1β causes its effects on electrolyte transport, the colonic tissue was treated with either indomethacin (1 μM), a cyclooxygenase inhibitor which prevents the production of prostaglandins, atropine (1 μM), a cholinergic inhibitor, or TTX (1

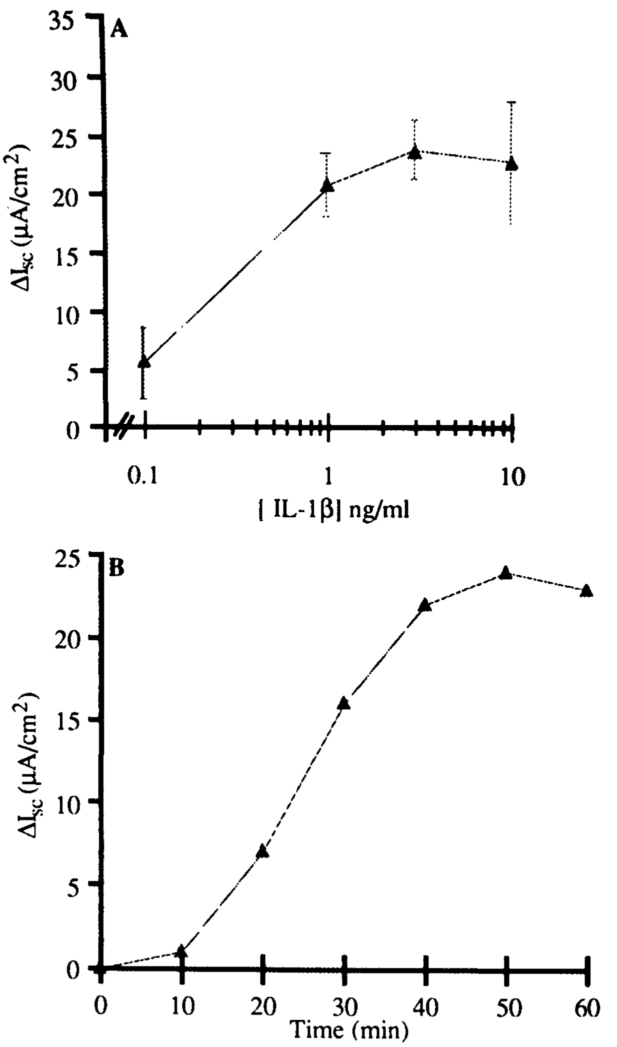

FIG. 1. (A) Concentration-dependent response of the effect of IL-1β on rabbit colonic short circuit current (*n* = 6). IL-1β was added to the serosal surface of the tissue, and the data based on the maximum increase in short-circuit current is shown for each concentration tested. A separate piece of stripped distal colon was used for each determination. (B) The effect of IL-1β on short-circuit current as a function of time from a single representative experiment.

μM), a neuronal $Na^+$ channel inhibitor which prevents neural input to the epithelial cells. IL-1β (1 ng/ml) was added to the serosal side of the colon in the Ussing chamber after pretreating the tissue with either of these inhibitors and the peak change in $I_{sc}$ was measured. Indomethacin decreased the $I_{sc}$ by 7.3 ± 2.1 μA/cm². It completely abolished the effects of IL-1β on $I_{sc}$ (% $\Delta I_{sc}$ caused by IL-1β in the presence of indomethacin was −10.2 ± 13.2 μA/cm², Fig. 2) suggesting that IL-1β mediates most of its effects through the production of prostaglandins.

Atropine alone had no effect on $I_{sc}$ and had no effect on IL-1-induced increases in $I_{sc}$. TTX, which alone caused a decrease in $I_{sc}$ of 8.2 ± 0.7 μA/cm², inhibited the effect of IL-1β by 56 ± 17% (Fig. 2) indicating that while there is a large portion of the effect being mediated through enteric nerves, there is a possibility of a direct effect exerted on epithelial cells.

*The nature of the ion involved in IL-1 effect:* To determine the nature of the ion(s) whose transport is affected by IL-1 treatment, treatment of the tissue with amiloride, bumetanide or ion substitution studies were performed. Chloride ion was substituted for gluconate to determine if it is involved in the action of the cytokine. Amiloride, at 1 μM, an inhibitor of the apical $Na^+$ channel, had no effect on the IL-1β induced increase in $I_{sc}$ (14.3 ± 1.2 μA/cm² in control tissue *vs.* 14.3 ± 0.9 μA/cm² in tissue treated with amiloride, $n = 3, p > 0.1$, NS), suggesting that $Na^+$ ion movement is not affected, while $Cl^-$ replacement completely reversed the increase in $I_{sc}$ (Fig. 3). Bumetanide (10 μM), an inhibitor of the $Na^+/K^+/2Cl^-$ transporter present on the basolateral membranes of the crypt cells, had no effect on $I_{sc}$ alone, also caused complete inhibition of the effect of IL-1β on $I_{sc}$ (16 ± 1 μA/cm² in control tissues *vs.* 0 ± 2 μA/cm² in bumetanide treated tissue, $n = 5, p < 0.005$; and −9 ± 3 μA/cm² in chloride-free buffer, $n = 5, p < 0.001$). From these data we conclude that IL-1β causes a significant increase in anion secretion, mainly $Cl^-$, which causes the increase in $I_{sc}$ observed in the Ussing chamber.

*The effect of IL-1β on the production of $PGE_2$:* Treatment of stripped tissue with IL-1β (1 ng/ml) caused a significant increase in the production of $PGE_2$ (Fig. 4) compared with a time control (5 ± 1 ng/mg protein in control tissue *vs.* 12 ± 2 ng/mg protein in treated tissue, $n = 6, p < 0.05$). The time course for $PGE_2$ production followed a similar course to the effect observed on $I_{sc}$ in Fig. 1b. The levels of $LTB_4$ were undetectable in control and in IL-1-treated tissues.

*Effects of IL-1β in the presence of cycloheximide and actinomycin D:* To test if protein synthesis is the reason for the delayed effect observed for the action of IL-1β, the tissue was treated with cycloheximide (100 μg/ml), a protein synthesis inhibitor, or with actinomycin D (10 μg/ml), a transcription inhibitor. As shown in Fig. 5, the effect of IL-1β on $I_{sc}$ was significantly inhibited when the tissue was treated with cycloheximide on the serosal side for 15 min prior to the addition of the cytokine. The IL-1β-induced increase in $I_{sc}$ in the presence of cycloheximide was 2.3 ± 1.2 μA/cm² *vs.* the effect of IL-1β alone, 16.3 ± 5.0 μA/cm² ($n = 5, p < 0.001$). Actinomycin D also caused an inhibition but this was not complete (Fig. 5). The IL-1β-induced increase in $I_{sc}$ in the presence of actinomycin D was 4.8 ± 2.8 μA/cm² *vs.* the effect of IL-1β alone, 16.4 ± 3.1 μA/cm² ($n = 5, p < 0.01$). This is attributed to the short time of incubation (2 h) with the inhibitor since the tissue's conductance started to decrease after 3–4 h of

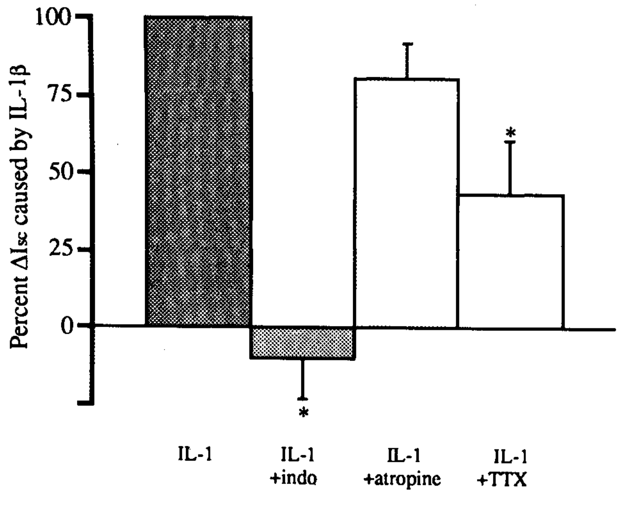

FIG. 2. The effect of IL-1β on short-circuit current alone and after incubating the tissue with 1 μM indomethacin (indo), 1 μM atropine and 1 μM TTX. The data (mean ± S.E., $n = 6$, *$p < 0.05$, $p$ value represents the comparison of the IL-1 on short-circuit current in the presence of the inhibitor to its effect alone) are presented as percentage change in short-circuit current caused by IL-1β.

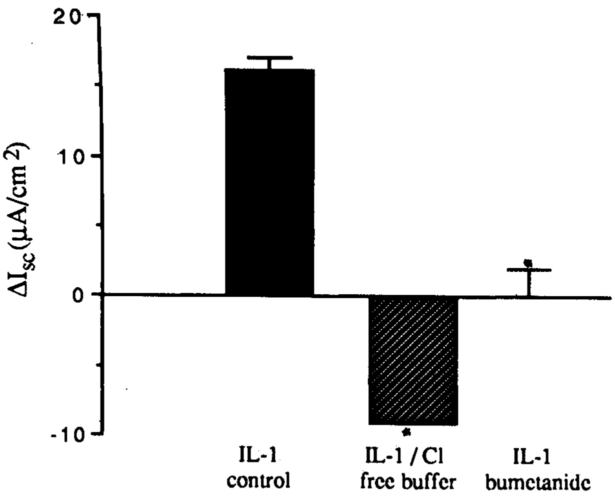

FIG. 3. The effect of IL-1β on short-circuit current alone and in the presence of chloride-free buffer or bumetanide (10 μM). The data (mean ± S.E., $n = 6$, *$p < 0.05$. $p$ value represents the comparison of the IL-1 on short-circuit current in the presence of the inhibitor to its effect alone) are presented as change in short-circuit current caused by IL-1β.

mounting in the Ussing chambers. Neither cycloheximide nor actinomycin D had any effect on $I_{sc}$ when added alone. In all these experiments, Br-cAMP (100 μM) was added at the end of the experiment to test if either of the treatments (cycloheximide or actinomycin D) were toxic to the tissue. Br-cAMP caused a rapid increase in $I_{sc}$ of 29.0 ± 5.7 μA/cm² in control tissue compared with 29.0 ± 0.9 μA/cm² ($p$ > 0.1, NS, $n$ = 5) in tissue treated with cycloheximide and 21.8 ± 3.2 μA/cm² in tissue treated with actinomycin D ($p$ > 0.1, NS; $n$ = 5). These data suggest that indeed protein synthesis is involved in the effect of IL-1β on ion transport and would explain the delayed effect observed in $I_{sc}$.

*The effect of IL-1β on the production of PGE₂ in the presence of cycloheximide:* Cycloheximide (100 μg/ml) significantly inhibited the increase in $PGE_2$ observed in tissue treated with IL-1β (Fig. 6). This suggests that IL-1β causes an increase in protein synthesis whose function is to increase the production of $PGE_2$ which in turn causes an increase in $Cl^-$ secretion. Cycloheximide had no significant effect on $PGE_2$ production in control untreated tissue.

*The effect of IL-1β in the presence of nitric oxide synthase (NOS) inhibitor:* To test if NO is mediating the effects of IL-1 in the rabbit distal colon, *NG*-nitro-L-arginine methyl ester (NAME) at different concentrations (0.5–5 Mm) was added to the serosal surface of the tissue and the effect of IL-1β on $I_{sc}$ was tested. The increase in $I_{sc}$ caused by IL-1β was not affected by NAME added either at the same time with the IL-1β or 20 min before ($I_{sc}$ = 11.5 ± 1.3 μA/cm² in control tissue *vs.* 9.2 ± 1.8 μA/cm² in tissue pretreated with NAME (0.5 mM) for 20 min prior to IL-1 addition, *vs.* 8.2 ± 2.1 μA/cm² in tissue treated with NAME and IL-1β simultaneously, $n$ = 5; $p$ > 0.1; NS). Similar results were obtained with the different concentrations of NAME used (data not shown).

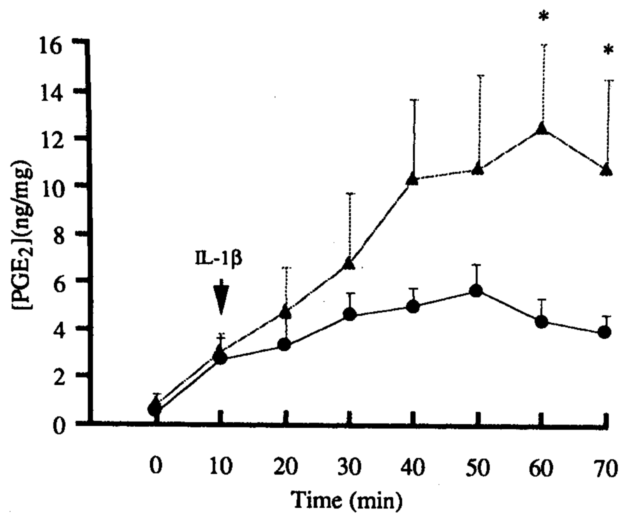

FIG. 4. The effect of IL-1β on the production of $PGE_2$ (▲) as a function of time, compared with a time control (●). Each point represents mean ± S.E., $n$ = 6, *$p$ < 0.05.

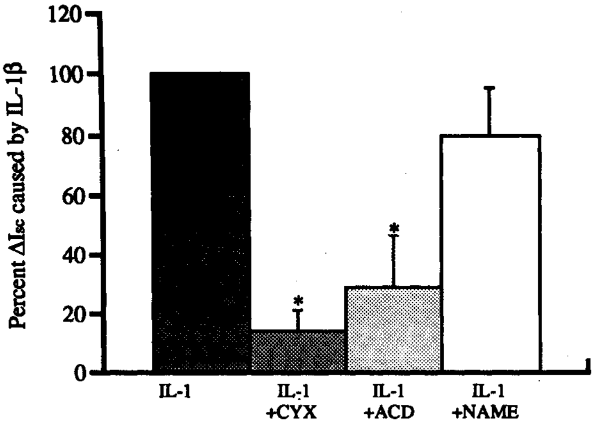

FIG. 5. The effect of IL-1β on short-circuit current alone and in the presence of cycloheximide (CYX, 100 μg/ml), actinomycin D (ACD, 10 μg/ml) and NAME (500 μM). The data (mean ± S.E., $n$ = 5, *$p$ < 0.05. $p$ value represents the comparison of the IL-1 on short-circuit current in the presence of the inhibitor to its effect alone) are presented as percentage change in short-circuit current caused by IL-1β.

*The effect of TNFα on $I_{sc}$ in rabbit distal colon:* We found no evidence in the rabbit colon for a similar action of TNFα to that observed by IL-1. TNF at different concentrations (0.1–100 ng/ml) added to the serosal bathing solution, had no effect on $I_{sc}$ (TNFα at 100 ng/ml, caused a change in $I_{sc}$ = –2.3 ± 1.2 μA/cm², $n$ = 3, not significantly different to zero) in rabbit distal colon (data not shown).

### Discussion

Cytokines such as IL-1, possess a broad spectrum of biological activity. They are now being recognized as essential mediators of both normal and pathological immune responses such as temperature regulation, bone and cartilage remodelling, and regulation of extracellular matrix products. However, increased levels of these cytokines can lead to predominance of proinflammatory effects that characterize disease states such as sepsis and inflammatory bowel disease, and to the generation of inflammatory mediators such as leukotriene $B_4$ or PGEs.[19] In this study it is reported that IL-1β stimulates ion secretion through a mechanism which involves the production of $PGE_2$. The increase in $PGE_2$ production observed in this

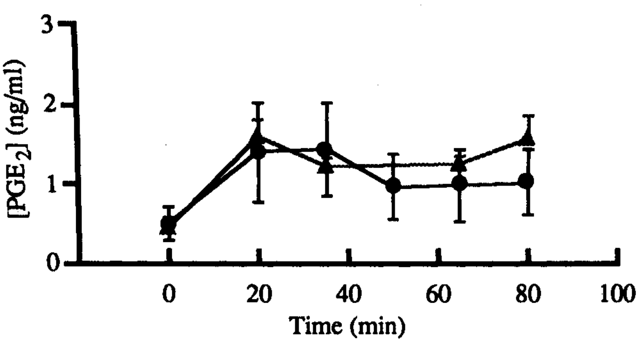

FIG. 6. Levels of IL-1 induced-production of $PGE_2$ in the presence of cycloheximide (100 μg/ml) (▲) compared with a time control (●). Each point represents mean ± S.E., $n$ = 3.

study is consistent with the *in vivo* experiments reported by Cominelli *et al.*[20] In the latter report, it was shown that in the isolated rabbit distal colon a 10 h infusion of IL-1 progressively increases production of $PGE_2$, 6-keto $PGF_{1\alpha}$ and thromobxane $B_2$. In the present study it was found that the effect of IL-1 on $I_{sc}$ and on $PGE_2$ production were not only concentration- and time-dependent but required protein synthesis. IL-1β caused an increase in $I_{sc}$ mainly by increasing $Cl^-$ secretion significantly with no effect on $Na^+$ ion movement through the apical $Na^+$ channel. In pancreatic islet cells, IL-1 was shown to activate the $Na^+/H^+$ exchanger and cause a rapid increase in cytosolic $Na^+$ concentration.[21] Since there is no evidence for the presence of the $Na^+/H^+$ exchanger in rabbit distal colon, the only possible explanation for the increased $I_{sc}$ is an increase in $Cl^-$ secretion.

Most of the effects of IL-1β on $I_{sc}$ and $PGE_2$ production were found to be through protein synthesis. Induction of protein synthesis in response to IL-1 treatment has been reported in a variety of cells.[22–25] In these studies, alterations in phospholipid metabolism were observed. These were primarily due to increased $PLA_2$ activity which occurred in response to IL-1 and which was attributed to induced synthesis of a phospholipase $A_2$ activating protein.[26] In human umbilical vein endothelial cells, IL-1 has recently been shown to induce cyclooxygenase-2 expression through a post-transcriptional regulation.[27] In insulin-secreting cells IL-1 modulates the expression of specific proteins, not yet identified, but might represent heat shock protein, superoxide dismutase, or other proteins involved in the functional response of the islet to oxidative stress and free radical formation.[28]

Although the cell surface receptor for IL-1 has been cloned,[29] it is still largely unknown how it transmits information to the inside of the cell after IL-1 binding. The intracellular signals induced by IL-1 appear to be complex because IL-1 seems to use multiple signal transduction pathways.[14,15] In the beta cell, for example, nitric oxide (NO) has been implicated as the effector molecule responsible for some of the effects of IL-1 on cell function. *NG*-monomethyl-L-arginine (NMMA) and NAME, both of which are competitive inhibitors of nitric oxide synthase, prevent the inhibitory effects of IL-1β on glucose-stimulated insulin secretion by isolated islets.[30] In our study, however, using a NOS inhibitor, we found no evidence for NO being a mediator for IL-1 action.

More than one second messenger seems to be involved in mediating the effects of IL-1. In different cell types, such as islets, pituitary, mesangial cells, chondrocytes and fibroblasts, different mediators have been described in association with cytokine action. Some of these mediators included $Ca^{2+}$, inositol phosphates, cAMP, protein kinase A, PLC and $PLA_2$ as well as alteration in gene transcription.[24,31–36] In the intestine, the second messenger(s) involved in mediating the effect(s) of IL-1 is yet to be determined.

The human intestinal mucosa has been shown to be a rich source of IL-1 under normal conditions and that production of both forms, IL-1α and IL-1β, is dramatically increased during pathological conditions such as inflammation.[6] IL-1 has been found to originate from mononuclear cells in the lamina propria but not from epithelial cells in normal or inflamed mucosa. Whether the receptor for IL-1 is present on epithelial cells, or not, is not yet known, although some evidence has emerged that IL-1β and IL-6 receptors have been identified on human epithelial cells suggesting that these cells can be regulated directly by proinflammatory cytokines.[37]

In conclusion, this study demonstrates that IL-1 has profound effects on the secretory function of the normal distal colon. Since IL-1 is significantly increased in inflammatory bowel disease, an understanding of its mechanism of action on electrolyte transport in the colon will lead to greater insights into the pathophysiology of inflammatory bowel disease and possibly new therapeutic modalities.

### References

1. Dinarello CA. Biology of interleukin 1. *FASEB J* 1988; **2**: 108–115.
2. Larrick JW, Kunkel SL. The role of tumor necrosis factor and interleukin 1 in the immunoinflammatory response. *Pharm Res* 1988; **5**: 129–139.
3. Saklatvala J, Guesdon F. Interleukin 1 and tumor necrosis factor signal transduction mechanisms: potential targets for pharmacological control of inflammation. *J Rheumatol* 1992; **19** (suppl): 65–69.
4. March CJ, Mosley B, Larsen A, *et al.* Cloning, sequence and expression of two distinct human interleukin-1 complementary DNAs. *Nature* 1985, **315**: 641–647.
5. Arai KI, Lee F, Miyajima A, Miyatake S, Arai N, Yokota T. Cytokines: coordination of immune and inflammatory responses. *Annu Rev Biochem* 1990; **59**: 783–836.
6. Ligumsky M, Simon PL, Karmeli F, Rachmilewitz D. Role of interleukin 1 in inflammatory bowel disease; enhanced production during active disease. *Gut* 1990; **31**: 686–689.
7. Mahida YR, Wu K, Jewell DP. Enhanced production of interleukin 1-β by mononuclear cells isolated from mucosa with active ulcerative colitis of Crohn's disease. *Gut* 1989; **30**: 835–838.
8. Youngman KR, Simon PL, West GA, *et al.* Localization of intestinal interleukin 1 activity and protein gene expression to lamina propria cells. *Gastroenterology* 1993; **104**: 749–758.
9. Burakoff R, Zhao L, Joseph I, Donovan V. The immunosuppressive agent FK506 prevents colitis and attenuates inflammatory mediator levels and myoelectric changes in a trinitrobenzene sulfonic acid model of colitis. *Gastroenterology* 1992; **102**: A599.
10. Cominelli F, Nast CC, Clark BD, *et al.* Interleukin (IL-1) gene expression, synthesis, and effect of specific IL-1 receptor blockade in rabbit immune complex colitis. *J Clin Invest 1990*; **86**: 972–980.
11. Rachmilewitz D, Simon PL, Schwartz LW, Griswold DE, Fondacaro JD, Wasserman MA. Inflammatory mediators of experimental colitis in rats. *Gastroenterology* 1989; **97**: 326–337.
12. Chang EB, Musch MW, Mayer L. Interleukins 1 and 3 stimulate anion secretion in chicken intestine. *Gastroenterology* 1990; **98**: 1518–1524.
13. Chiossone DC, Simon PL, Smith PL. Interleukin-1: effects on rabbit ileal mucosal ion transport *in vitro*. *Eur J Pharmacol* 1990; **180**: 217–228.
14. Mizel SB. Cyclic AMP and interleukin 1 signal transduction. *Immunol Today* 1990; **11**: 390–391.
15. O'Neil LAJ, Bird TA, Saklatvala J. How does interleukin 1 activate cells? Interleukin 1 signal transduction. *Immunol Today* 1990; **11**: 392–394.
16. Simon PL, Lee JC. Evidence for shared antigenic determinants on rabbit and human interleukin 1 (IL 1). *J Immunol* 1986; **137**: 557–562.
17. Furutani Y, Notake M, Yamayoshi M, *et al.* Cloning and characterization of the cDNA for human and rabbit interleukin-1 precursor. *Nucleic Acid Res* 1985; **13**: 5869–5882.
18. Segel IH. *Biochemical Calculations.* New York: Wiley, 1976: 236.
19. Standiford TJ, Strieter RM. TNF and IL-1 in sepsis: good cytokines gone bad. *J Lab Clin Med* 1992; 179–180.
20. Cominelli F, Nast CC, Dinarello CA, Gentilini P, Zipser RD. Regulation of eicosanoid production in rabbit colon by interleukin-1. *Gastroenterology* 1989; **97**: 1400–1405.

21. Sandler S, Eizirik DL, Svensson C, Strandell E, Welsh M, Welsh N. Biochemical and molecular actions of interleukin-1 on pancreatic β-cells. *Autoimmunity* 1991; **10**: 241–253.
22. Bomalaski JS, Clark MS. Activation of phospholipase $A_2$ in rheumatoid arthritis. *Adv Exp Med Biol* 1990; **279**: 231–238.
23. Crowl RM, Stoller TJ, Conroy RR, Stoner CR. Induction of phospholipase $A_2$ gene expression in human hepatoma cells by mediators of the acute phase response. *J Biol Chem* 1991; **266**: 2647–2651.
24. Gilman SC, Chang J, Zeigler PR, Uhl J, Mochan E. Interleukin-1 activates phospholipase $A_2$ in human synovial cells. *Arthritis Rheum* 1988; **31**: 126–130.
25. Godfrey RW, Johnson WJ, Newman T, Hoffstein ST. Interleukin-1 and tumor necrosis factor are not synergistic for human synovial fibroblast $PLA_2$ activation and $PGE_2$ production. *Prostaglandins* 1988; **35**: 107–114.
26. Bomalaski JS, Steiner MR, Simon PL, Clark MA. IL-1 increases phospholipase $A_2$ activity, expression of phospholipase $A_2$-activating protein, and release of linoleic acid from murine T helper cell line EL-4. *J Immunol* 1992; **148**: 155–160.
27. Ristimaki A, Garfinkel S, Wessendorf J, Maciag T, Hla T. Induction of cyclooxygenase-2 by interleukin-1α. Evidence for post-transcriptional regulation. *J Biol Chem* 1994; **269**: 11769–11775.
28. Helqvist S, Hansen BS, Johannesen J, Andersen HU, Nielsen JH, Nerup J. Interleukin 1 induces new protein formation in isolated rat islets of Langerhans. *Acta Endocrinologica* 1989; **121**: 136–140.
29. Sims JE, March CJ, Cosman D, *et al.* cDNA expression cloning of the IL-1 receptor, a member of the immunoglobulin superfamily. *Science* 1988; **241**: 585–589.
30. Corbett JA, Wang JL, Sweetland MA, Lancaster Jr JR, McDaniel ML. Interleukin 1β induces the formation of nitric oxide by β-cells purified from rodent islets of Langerhans. Evidence for the β-cell as a source and site of action of nitric oxide. *J Clin Invest* 1992; **90**: 2384–2391.
31. Argiles JM, Lopez-Soriano J, Ortiz MA, Pou JM, Lopez-Soriano JF. Interleukin-1 and b-cell function: more than one second messenger? *Endocrine Rev* 1992; **13**: 515–524.
32. Chang J, Gilman SC, Lewis AJ. Interleukin-1 activates phospholipase $A_2$ in rabbit chondrocytes: a possible signal for IL-1 action. *J Immunol* 1986; **136**: 1283–1287.
33. Gwosdow AR, O'Connell NA, Abou-Samra AB. Interleukin-1 increases protein kinase A activity by a cAMP-independent mechanism in AtT-20 cells. *Am J Physiol* 1994; **266**: E79–E84.
34. Nakazato Y, Simonson MS, Herman WH, Konieczkowski M, Sedor JR. Interleukin-1a stimulates prostaglandin biosynthesis in serum-activated mesangial cells by induction of a non-pancreatic (type II) phospholipase $A_2$. *J Biol Chem* 1991; **266**: 14119–14127.
35. Pfeilschifter J, Schalkwijk C, Briner VA, van den Bosch H. Cytokine-stimulated secretion of group II phospholipase $A_2$ by rat mesangial cells. Its contribution to arachidonic acid release and prostaglandin synthesis by cultured rat glomerular cells. *J Clin Invest* 1993; **2**: 2516–2523.
36. Takii T, Akahoshi T, Kato K, Hayashi H, Marunouchi T, Onozaki K. Interleukin-1 up-regulates transcription of its own receptor in a human fibroblast cell line TIG-1: role of endogenous $PGE_2$ and cAMP. *Eur J Immunol* 1992; **22**: 1221–1227.
37. Panja A, Goldberg S, Mayer L. Expression and regulation of cytokine receptors on intestinal epithelial cells from normal controls and patients with IBD. *Gastroenterology* 1993; **972**: A243.

**Received 1 September 1994; accepted in revised form 19 October 1994**
